# Stress and burnout: exploring postgraduate physiotherapy students’ experiences and coping strategies

**DOI:** 10.1186/s12909-020-02360-6

**Published:** 2020-11-16

**Authors:** Tess Brooke, Makaela Brown, Robin Orr, Suzanne Gough

**Affiliations:** 1 Faculty of Health Sciences and Medicine, Bond University, 2 Promethean Way, Robina, Gold Coast, QLD 4226 Australia; 2 Tactical Research Unit, Bond University, 2 Promethean Way, Robina, Gold Coast, QLD 4226 Australia

**Keywords:** Pre-registration physiotherapy students, Perceived stress, Perceived burnout, Coping strategies, Postgraduate

## Abstract

**Background and purpose:**

The impact of stress and burnout in students is an emerging topic. When students perceive that they are unable to cope with stressors, there is increased potential for burnout. To maximise students’ higher educational institution outcomes, students must be able to effectively cope with stressful demands. Research suggests physiotherapy students, in particular, suffer from a high risk of stress and burnout, however limited research exists on postgraduate, pre-registration, physiotherapy students. The purpose of this study was to determine perceived stress, burnout and associated coping strategies across three timepoints in the first year of a postgraduate, pre-registration physiotherapy program.

**Methods:**

A qualitative and quantitative survey design was utilised at one Australian Higher Education Institution. The 51-item self-administered questionnaire consisted of demographics, the Coping Self Efficacy (CSE) Scale and Maslach Burnout Inventory – General Survey for Students (MBI-GS (S)), and open-ended questions. The questionnaire was administered at three timepoints (T) in the program: T1 at the start of semester 1, T2 before the first placement in semester 2 and T3 after 10-weeks of placement. Data were analysed using descriptive, statistical and thematical analysis.

**Subjects:**

All first year Doctor of Physiotherapy students.

**Results:**

A response rate of 62% (*n* = 38) was achieved. There were no differences in stress and burnout scores between sexes, nor differences in stress and burnout over time. Highest median CSE scores were seen at T1, with highest median MBI-GS (S) cynicism scores at T2, exhaustion (EX) at T1 and T2, and professional efficacy at T1 and T2. The greatest mean CSE changes were seen from T1-T2 and T1–3, and PE greatest changes from T2-T3 and T1-T3. No strong correlation was found between stress and burnout. Curriculum coursework was a frequently reported stressor, along with clinical placement and transition periods. Coping strategies utilized by students were both positive and maladaptive. Positive strategies included sporting activities, baking, listening to music, and social connections, whereas maladaptive strategies included alcohol consumption, excessive eating, and gaming.

**Conclusion:**

Student consistently identified periods of stress and burnout, with curriculum coursework in particular being a trigger. Findings acknowledge the need for further investigation on sources of perceived stress, burnout, and coping mechanisms to optimise student welfare and enhance Higher Education Institution outcomes.

## Background

Stress is defined as the dynamic interaction encompassing the physical, emotional, and psychological responses between the individual and their environment [1]. Stress has continually been reported to be influenced by higher educational demands within pre-registration (Bachelors, Masters and Doctor of Physiotherapy) physiotherapy programs [2–11]. In addition, stress is also affected by other external factors including relationships, relocation, and financial pressures [12]. Previous studies [2, 9, 12] have reported that the leading cause of stress in pre-registration physiotherapy students is academic stress, followed by social related stressors (e.g. spending time on extra-curricular activities, or time with friends and family). Previous studies conducted globally have explored relationships between physiotherapy coursework and perceptions of student stress [2–4, 7–9, 12–22], stress and associated coping strategies [4–6, 10, 11, 14–16], sources of stress perceived course difficulty and paid employment [12], or stress associated with simulation and hospital based clinical education [17].

When physiotherapy students perceive that they are unable to cope with stressful demands, the impacts of the stressors may manifest as burnout [1]. Burnout is considered to be a multifaceted behavioural syndrome often leading to negative responses for an individual when exposed for a prolonged period of time [23]. Burnout can affect an individual’s physiological and psychopathological system causing anxiety, depression, interpersonal sensitivity, alcoholism, and other addictive disorders [21]. Previous research by Balogun et al. [24] found that emotional exhaustion and depersonalization scores in physiotherapy students were higher than reports for normative data within the majority of service professionals. Noting this potential risk of burnout in pre-registration physiotherapy programs, identifying whether students are capable of implementing coping strategies is of importance.

Burnout has been well documented amongst qualified physiotherapists [25–31]. In 1995, Scutter and Goold [32] reported the presence of burnout in recently qualified physiotherapists. Whereas, in 1994, O’Meara and colleagues [33] and Kolb [34] suggested that burnout begins during physiotherapy education, well before establishment of a career or specialisation. Whilst Balogun and colleagues [24] explored burnout in a single academic semester, they also established that academic performance is not a viable determinant of physiotherapy students [35]. To date, there remains a paucity of literature exploring the causes of burnout in physiotherapy education.

Individuals have differing perceptions of stress and as a consequence utilize different coping strategies [6]. Coping strategies can be defined as the ability to adapt in response to a range of events that are perceived as stressful [36]. A study by Kohn, et al. [36] suggests that three general strategies exist in an attempt to cope with situations that are stressful; 1) problem-focused coping, which aims to remedy the threatening situation; 2) emotion-focused coping, which aims to manage the response; and 3) avoidance-focused coping, which involves attempts to remove the threatening situation. While differences exist between coping perceptions over time, along with an increased ability to positively respond to stressful situations [11]. Pierceall et al. [10] suggest the following as commonly successful coping mechanisms: talking to family and friends, leisure activities, and exercising. However, despite implementation of positive coping strategies, maladaptive coping strategies commonly arise, including alcohol abuse, drug abuse and smoking.

Current research identifies challenges and imposing demands placed on physiotherapy students and the subsequent impacts both psychologically and emotionally that these demands have [2, 3, 11]. To date, the impact of stress and burnout in physiotherapy students across the first year of study has not been explored. The ability to cope with stress is an important factor affecting a DPHTY student’s success in an academic program [11]. Syed and colleagues [37] reported the frequency of depression, anxiety and stress was 48, 69 and 53% respectively, amongst undergraduate physiotherapy students in Pakistan [37]. Stress and burnout can furthermore affect students’ emotional wellbeing and can result in psychological morbidity [38]. Noting these concerns, there are differences in the structure of physiotherapy programs with some programs being conducted in an intensive fashion. For example, the Doctor of Physiotherapy (DPHTY) program at Bond University in Australia, a postgraduate entry-level physiotherapy program, comprises six semesters over 2 years (three trimesters per year), totaling 18 subjects and over 1200 clinical placement hours (50% of the program) [39]. How first year physiotherapy students in an intensive, postgraduate course perceive, and experience stress and their coping strategies may differ from those completing the degree over several years and / or completing their first degree. The aims of this study were to identify the complexity of factors that influence first year Doctor of Physiotherapy (DPHTY) students’ perceptions of stress, burnout and coping strategies with the intent of answering three research questions being: 1) Was there a difference in perceived stress and burnout over time?; 2) Did sex influence the amount of stress and burnout experienced?; and 3) What were the coping mechanisms utilized by pre-registration DPHTY students?

## Methods

### Design

A qualitative/quantitative survey design was utilised. To achieve the aims this study a self-reported survey instrument was developed to explore postgraduate physiotherapy students’ perceptions of stress, burnout and coping strategies as they experienced various transitions between coursework and clinical placements and vice-versa, during the first year of the curriculum.

### Instrumentation

A self-reported 51-item mixed methods questionnaire was specifically designed to address the aim of the study and research questions. The survey consisted of four sections: demographic questions, two validated questionnaires, the Coping Self-Efficacy Scale (CSE) [40] and the Maslach Burnout Inventory-General Survey for Students (MBI-GS (S) [41] generating quantitative data, and open-ended personal reflection questions.

The CSE is a valid and reliable tool with a reported intra-class correlation coefficient of *p* < 0.001 and criterion validity of *p* < 0.001 [42]. The CSE scale was incorporated to measure an individual’s perceived ability to cope effectively with life challenges, and to assess changes in CSE scores [40]. The MBI-GS has also been proven to be a reliable and valid tool. There are no current reports on psychometric properties in the student version MBI-GS (S) hence the use of the general version MBI-GS reliability and validity scores [43]. The MBI-GS (S) was selected for inclusion in the questionnaire as it is recognized as the leading measure of burnout in students at college and university [43]. Additionally, open-ended questions allowed for qualitative responses to be gathered on periods of stress encountered in the current semester, coping strategies that have been successful or unsuccessful during the current semester, further information not covered in previous questions, and three things the students wish they had known before starting the current semester and why.

This bespoke questionnaire was developed by the authors who had experience in curriculum design, delivery, assessment and clinical placement support across Bachelors with Honours, Masters and Doctoral pre-registration and postgraduate physiotherapy programs in the UK and Australia. Electronic distribution was not possible when combining validated tools.

### Pilot study

A pilot study was carried out to assess the accuracy and clarity of the instructions for completion and wording of the questions. The questionnaire was piloted by second year DPHTY students, who were excluded for participation in the main study. The questionnaire was designed and piloted to ensure that no harm would arise from participation in the survey. The pilot established the questionnaire completion time of up to 15–20 min. It was anticipated the time would be reduced if students participated in subsequent timepoints as the questions were identical.

### Setting and participants

The DPHTY program is an intensive 6 semester extended masters’ degree, which embeds a problem-based learning (PBL) model within coursework subjects alongside clinical skills and simulation-based education learning activities, evidence-based practice research subjects and over 1200 h of clinical placements [39].

All 61 students enrolled in their first year of a postgraduate pre-registration, intensive DPHTY were invited to participate by a Research Assistant. The questionnaire was administered at three timepoints (T) in the program: T1 at the start of the first semester 1, T2 immediately before the first placement in semester 2 and T3 following completion of 10-weeks of placement.

Gatekeeper approval was sought prior to inviting student to participate. All students were invited to participate in the survey each timepoint, irrespective of previous participation at any timepoint. Students were invited to participate by a Research Assistant at the end of an on-campus timetabled activity, who was not part of the academic team, nor the data analysis.

### Data analysis

Raw data was entered into the IBM Statistical Package for the Social Sciences, Version 26.0 (IBM Corp, 2019 [44], by the Research Assistant and verified by two members of the team (TB and MB). Descriptive statistics were used to summarise and present quantitative data. All quantitative data were assessed for normality using the Shapiro-Wilk test. Where a lack of normal distribution was found, medians and ranges were reported. A Fisher’s Exact test was conducted to assess the significance of each variable, with a significance level of *p* < 0.05. As different students were represented in different paired groups (e.g. T1 and T2 but not T3 or T2 and T3 but not T1) paired sample t-tests were used to investigate changes between shared timepoints. The paired samples t-test compared the mean total scores of stress and burnout at opposing timepoints (T1 versus T2, T2 versus T3 and T1 versus T3). A Mann Whitney U test was conducted to assess significance, with a *p*-value of ≤0.017 that accounts for multiplicity (0.05/3). Qualitative responses were subjected to a 5-step thematic analysis approach of data familiarisation, identification of a thematic framework (including integration of a priori themes) (including integration of a priori themes), indexing, charting, mapping and interpretation (by TB and MB). Comparisons were made between the qualitative data themes produced and modifications agreed with a third reviewer (SG), to create the final themes, which aimed to reduce reviewer bias and generate rigorous analysis [45]. Thematic framework analysis [46] was selected as the most appropriate method for this study, ensuring the relationship between the context and content could be adequately presented [46]. Qualitative and quantitative data were given equal priority.

## Results

### Demographic characteristics of participants

Of the 61 DPHTY first year students invited to participate in the study, 38 (62%) participated across any of three timepoints. Of these respondents, 27 (71%) responded at T1, 30 (79%) responded at T2, and 20 (53%) responded at T3. The median age of students *n* = 38) was 24.0 years of age, ranging from 20.0 to 38.0 years. Demographics of the student population are detailed in Table 1.

**Table 1:** Cohort median and paired samples t-test results for CSE total scores and MBI scores

Outcome measure	Median (range)	Total/Subscale (Time point)	Mean difference	(95% CI)	Paired diff. ***p***-value
T1 CSE	170.0 (79.0–254.0)	CSE –Total (T1–T2)	−9.2	(−27.9, 9.5)	0.318
T2 CSE	165.5 (88.0–258.0)	CSE – Total (T2-T3)	0.6	(−11.9, 13.2)	0.914
T3 CSE	162.5 (95.0–260.0)	CSE – Total (T1-T3)	−8.8	(−27.5, 9.9)	0.330
T1 MBI CYN	1.0 (0.0–4.0)	CYN T1-T2	−0.5	(−1.1, 0.2)	0.135
T2 MBI CYN	2.0 (0.0–4.0)	CYN T2-T3	0.7	(−0.0, 1.3)	0.055
T3 MBI CYN	1.0 (0.0–6.0)	CYN T1-T3	−.02	(−1.0, 0.6)	0.628
T1 MBI EX	4.0 (1.0–5.0)	EXH T1-T2	−0.2	(−1.0, 0.6)	0.565
T2 MBI EX	4.0 (0.0–6.0)	EXH T2-T3	1.1	(0.1, 2.1)	0.040
T3 MBI EX	3.5 (0.0–6.0)	EXH T1-T3	−0.2	(−1.1, 0.8)	0.682
T1 MBI PE	5.0 (2.0–6.0)	PE T1-T2	1.3	(−2.5, 5.2)	0.482
T2 MBI PE	5.0 (0.0–6.0)	PE T2-T3	4.7	(−1.5, 10.8)	0.128
T3 MBI PE	4.0 (0.0–6.0)	PE T1-T3	3.1	(−1.8, 8.0)	0.194

Significance *p*-value ≤ 0.017 (accounted for multiplicity 0.05/3). Negative mean differences indicate smaller scores at the corresponding time point

Key: *CSE* Coping self efficacy scale, *MBI-GS (S)* Maslach Burnout Inventory – General Survey (Students), *T1* Time point 1 start of course, *T2* Time point 2 prior to clinical placement, *T3* Time point 3 immediately post placement, *CYN* Cynicism subscale, *EXH* Exhaustion subscale, *PE* Professional Efficacy subscale

### Quantitative results

In general, the results show that all students reported feeling some level of stress and burnout in their responses. The results of the paired t-tests used to answer the first research question (Was there a difference in perceived stress and burnout over time?) is presented in Table 1. CSE scores showed the greatest mean changes from T1-T2 and T1-T3, however these differences were not significant (Table 2). MBI-GS (S) mean scores identified that PE changes were greatest from T2-T3 and T1-T3, however were likewise not significant (Table 1).

**Table 2:** Demographic information and descriptive data for student CSE and MBI scores in relation to sex

Characteristic	Females (***n*** = 22)	Males (***n*** = 16)	***P***-value
Age (years), median (range)	23.0 (20.0–38.0)	26.5 (21.0–36.0)	0.012
Qualification, n (%)			0.370
Bachelor	18.0 (81.8%)	15.0 (93.8%)	
Masters	4.0 (18.2%)	1.0 (6.3%)	
T1 CSE	157.0 (79.0–220.0)	171.5 (97.0–254.0)	0.256
T2 CSE	159.0 (88.0–254.0)	177.5 (128.0–258.0)	0.185
T3 CSE	155.0 (95.0–222.0)	167.0 (125.0–260.0)	0.370
T1 MBI CYN	1.0 (0.0–4.0)	1.0 (0.0–4.0)	0.193
T2 MBI CYN	1.0 (0.0–3.0)	2.0 (0.0–4.0)	0.185
T3 MBI CYN	1.0 (0.0–5.0)	1.0 (0.0–6.0)	0.955
T1 MBI EX	4.0 (0.0–5.0)	3.5 (0.0–5.0)	0.427
T2 MBI EX	4.0 (1.0–6.0)	4.5 (0.0–6.0)	0.950
T3 MBI EX	4.0 (0.0–5.0)	3.0 (0.0–6.0)	0.392
T1 MBI PE	5.0 (2.0–6.0)	5.0 (4.0–6.0)	0.581
T2 MBI PE	5.0 (3.0–6.0)	5.0 (0.0–6.0)	0.415
T3 MBI PE	4.0 (0.0–6.0)	4.0 (0.0–6.0)	0.955

Significance: *p*-value ≤ 0.05

Key: *CSE* Coping Self Efficacy Scale, *MBI-GS (S)* Maslach Burnout Inventory – General Survey (Students), *T1* Timepoint 1 at the start of the first semester, *T2* Timepoint 2 refers to semester two, when the students were finalizing coursework subjects on campus, immediately prior to engaging in their first clinical placement, *T3* Timepoint 3 immediately after the completion of the first two consecutive five-week placement blocks, *CYN* Cynicism subscale, *EX* Exhaustion subscale, *PE* Professional Efficacy subscale

In relation to the second research question (Did sex influence the amount of stress and burnout experienced?), the median age of female participants was 23 (20–38) and for males was 26.5 (21–36) years, with strong evidence for significance between groups (*p* = 0.012) (Table 2). There was no statistically significant difference between groups in relation to their previous degree (Table 2).

Differences between sexes were observed across CSE scale and MBI-GS (S) subgroups, however these differences did not reach significance (Table 2). Highest median CSE scores for both males and females were seen at T1, and lowest scores at T3 (Table 1). The highest MBI-GS (S) Cynicism subscale median scores were reported at T2, Exhaustion subscale at T1 and T2, and professional Efficacy subscale at T1 and T2 (Table 1).

Of the 51 total responses across timepoints for the MBI-GS (S) question “I feel depressed by my studies”, 44 (86%) gave scores greater than zero, indicating that at some point during the timepoints, students have felt depressed by their studies.

### Qualitative results

Participants provided responses to the open-ended questions at each of the three timepoints T1 27/27, T2, 26/30 and T3 19/20 participants respectively). Answering the third research question (What were the coping mechanisms utilized by pre-registration DPHTY students?), the thematic analysis revealed common stress and burnout influencers and common coping strategies (Table 3). Thematic analysis revealed seven organizing themes across the three timepoints including: curriculum coursework, clinical placement, transition periods, work-life balance, coping behaviors, financial pressures, and course survival tips.

**Table 3:** Thematic analysis

Organizing Theme	Basic theme and time point prevalence	Exemplar Quotations
**1. Curriculum coursework**	1.1 Academic functioning^a^ challenges T1, T2	“Workload stress [leads to] feeling pressure/stress regarding amount of work to review and get done.” “I feel there aren’t enough hours in the day. We spend hours in class and then are expected to go home and study/do work. I feel stressed and overwhelmed when I don’t have enough time to go for a run or do something for myself.”
	1.2 Examination stress T1, T2, T3	“During the exam period where we had 3 exams in 3 days which were quite different from each other, week prior to the week of exams, after the exams where we are waiting for results of exams/ assessment.” “Exams were really stressful and overwhelming, I found my mental health deteriorated and I felt so much pressure to do well and know what I how much to study, I broke down a couple of times in the week or two leading up to the written exam.”
**2. Clinical placement**	2.1 Placement duration T3	“Having the placements before our holiday break was very helpful to solidify our learning. I loved the 10-week block of placements. I feel it will be nicer to have shorter blocks at class and then placements back to back, 10 and 10 is great. It’s a very stressful course.” “I feel it may be useful to have a few days break in between placements to have a good mental/physical break prior to starting the next block.”
	2.2 Placement stress T3	“I felt extremely stress on my first 5 weeks of my clinical placement. My educator hardly gave any immediate feedback and it made me very anxious as to knowing whether I was doing well or not. I got very unwell in my second block of my placement and I was physically/mentally exhausted making it difficult to perform to the best of my abilities.” “Being on placement, coming home and preparing an in-service presentation and reflection - very overloaded at end each time.”
**3. Transition periods**	3.1 Relocation adjustment T1, T3	“First week of class, it was a big adjustment moving from home and disruption of routine.” “The biggest stressor has been missing family and friends back home at the end of the semester.”
	3.2 Transition to academic life T1	“During the first week of classes it was overwhelming to be back at school again after 2 years, but I adapted quickly.” “During exam periods having back to back exams trying to balance sleep and stress of studying. Initially starting uni at bond.”
	3.3 Transition to placement T3	“Transitioning from placement 1 to 2 over a short period of time was quite stressful mentally and emotionally.”
	3.4 Transition doubt T3	“[I’m] questioning if this is really what I want to do because I haven’t enjoyed much of the course.”
**4. Work-life balance**	4.1 Lack of time management skills T1, T2	“Constantly thinking about how much I have to get done. Leads to anxiety attacks and poor sleep.” “How little time off I would have. How much university would cut into my social life.”
	4.2 Health impact and wellbeing T1, T2, T3	“I’ve been burnt out from last semester, which made me ill with the flu and I’ve had lingering flu ever since leaving me extra upset about situations and it has been affecting my relationship and my strong mindset.”
**5. Coping Behaviours**^**a**^	5.1 Study skill social support^b^ T1, T2	“Being with friends from cohort, discussing any uncertainties regarding study or content with friends, calling my family members regularly.” “Studying on campus rather than at home. Studying with others at times but also studying on my own at times.”
	5.2 Exercise/recreational activities T1, T2, T3	“I get up at 5.30 am every morning to exercise as I feel this is personal time for me to destress and do something for myself.” “I tried to maintain my exercise regime, but I believe it made me even more exhausted after really long days.” “Listen to music/podcast (not physio related)”
	5.3 Maladaptive behaviours T1, T2, T3	“Excessive drinking [alcohol]” “Stress eating to the extreme which has been unsuccessful”
	5.4 Mindfulness and positive self-talk^b^ T1, T3	“Trying to have positive thoughts on what I achieved so far and trusting ill get ‘through’ this” “Focus on end goal and the impact I can make in end”
	5.5 Religion/spiritual^b^ T3	“Pray and calm down mind” “Attending church every weekend to reignite my spirit”
	5.6 Psychological intervention T1, T3	“Saw psychologist week 6. Not feeling myself, felt brain couldn’t concentrate or process” “I visited a psychologist a few times whilst on placement and I believe this helped me to develop some coping strategies”
**6. Financial pressures**	6.1 Curriculum costs T1, T2, T3	“The travel to each of my placements was also very stressful as I was unable to afford extra accommodation in [X location] as I rent with my [name] on the [Y location]. It was 2.5 h each way every day” “Main periods of stress have been reassuring myself I can do what I want with this degree, that it’s not a waste of time/money and it was the best decision for me”
**7. Course survival tips**	7.1 Curriculum details T1, T2, T3	“I wish I knew what a typical day as a Bond University physiotherapy student looked like” “The amount of content that was going to be covered in a short period of time” “How much travel I would need to do for clinic”
	7.2 Self-management techniques T2, T3	“Take time for yourself so you don’t burn out. Learn as the semester goes.” “Best strategies for dealing with homesickness. Best way to focus on work is thinking about how close I was to going home. A little more about how working in a hospital really feels.”

Key: ^a^A priori themes from the Social and Academic Functioning (SAF) Scale, ^b^ A priori themes from the CSE scale

#### Theme 1: curriculum coursework

Curriculum coursework was an organizing theme that resonated across all timepoints. The basic theme ‘academic functioning challenges’ was evident throughout responses during T1 and T2. Students report not knowing where to focus or prioritize study, for example finding balancing study and study topics challenging. Another academic functioning challenge reported by students related to a lack of accent training and language barriers. For students on the DPHTY program English may be a second language, thus dialects and accents may add additional challenges to learning. Examination stress also appeared to be a prevalent basic theme where participants described increased stress especially with completing three exams in 3 days (Table 3).

#### Theme 2: clinical placement

In general, students described the 10-week clinical placement block as “enjoyable”, with reports that a few days break between the clinical placement block may be beneficial. Due to these responses arising in T3, potentially, students were less focused and less stressed about clinical placement during the earlier aspect of the program. Students reported feeling stressed about professional behaviours – receiving feedback, performance standards, self-directed learning and professional development (Table 3).

#### Theme 3: transition periods

Transitions to university life, clinical placement and relocating were reported by students in both T1 and T3, including difficulty with being away from friends and family, and difficulties with cohort cohesion. Students also reported doubts and questioning whether they want to continue the program due to not currently enjoying the course.

#### Theme 4: work-life balance

Work-life balance featured in students’ responses across all timepoints, specifically relating to difficulty finding the right balance between university life and having time for themselves. Students reported a lack of time management and guilt when doing activities other than study. Difficulties maintaining relationships and having time to be involved in social activities were also reported (Table 3).

#### Theme 5: coping strategies

Both positive and maladaptive coping strategies were evident across all three timepoints. Successful coping strategies commonly reported included social support coupled with study skills, exercise/recreational activities, and mindfulness and positive self-talk (Table 3). Whilst some students openly reported seeking psychological intervention in order to cope, others reported adopting maladaptive coping mechanisms including excessive alcohol consumption, excessive eating, and gaming.

#### Theme 6: financial pressures

Financial burden was reported across all three timepoints. Difficulties were reported especially whilst on placement, due to travel requirements and having to source accommodation (often resulting in paying multiple rent). Sourcing part-time work was reported to be difficult for students due to time pressures and course intensity (Table 3).

#### Theme 7: course survival tips

Students consistently reported that they wish they had known more curriculum details and self-management techniques throughout all three timepoints. They wish they had known more information on content delivery along with a typical day as a DPHTY university student. Participants also reported that they needed to take time to themselves so to avoid burnout, with other reports stating that they wish they had known the best strategies for dealing with homesickness (Table 3).

## Discussion

This study explored pre-registration DPTHY students’ levels of perceived stress, burnout, and associated coping mechanisms. The first research question focused on differences in perceived stress and burnout over time. The quantitative data from the CSE and MBI-GS (S) scores showed no significant changes at timepoints and between timepoints. These results are in contrast to previous research. A previous study reported by Van Veld et al. [11], conducted in the United States, found changes in CSE scores over time in first year DPHTY students. This difference between studies could be due to a variety of factors including timing of placement blocks, the curriculum coursework demands as well as the structure of the different programs.

Findings relating to the MBI-GS (S) are unique to this study, as it is the first study completed on physiotherapy students to utilise this version of the MBI. Previous studies [24, 35] have utilized the MBI, however an alternate version has meant the subscales differ from those within the current study. Balogun et al. [35] completed a study in 1996 that determined that academic performance was not correlated with physiotherapy students’ perceived levels of burnout. In another study, the same authors [24] found the emotional exhaustion and depersonalisation scores for physiotherapy students, were higher than the norms reported for the general population and most human services professionals. Whilst there is a paucity of research on the relationship between burnout and physiotherapy students, previous research has identified that if qualified physiotherapists are experiencing burnout, they may be less productive and engaged in their work, leading to poorer efficiency and patient satisfaction [30]. Raising awareness of stress, burnout, and coping strategies at the commencement of a physiotherapy program may facilitate greater self-awareness and self-management. It has been suggested that the incidence of burnout syndrome in qualified physiotherapists is similar to normative data observed in other health and medical related professions [31].

Qualitative data suggests that changes in written responses in regard to the type of stressors across the timepoints could have been associated with key transition periods associated with higher education. Difficulties found within transition periods including relocation adjustments, transition to academic life, transition to placement, and transition doubt, support the findings of previous studies [19]. Findings of the current study indicate a strong correlation between stress scores at the start of the course (T1) and pre-placement (T2). These results could have been due to similarities in stress perceptions and coping at these timepoints.

A strong theme throughout the qualitative responses was that curriculum coursework and examinations caused heightened levels of stress. Students reported periods of stress associated with coursework and course load commitments, which is consistent with previous research [8, 11–13, 16, 21, 24] conducted on undergraduate and pre-registration postgraduate students in several countries including Australia, USA, Israel and Sweden. Students frequently reported that their social life was lacking, along with time to undertake their hobbies due to workload and demands of the program. Students are required to undertake written examinations, seminar presentations, and practical examinations (OSCEs). Participants within the study reported feeling underprepared and anxious in the week/s leading up to assessments. A previous cross- sectional study [12] conducted in Australia and the United Kingdom found coursework stress to be the greatest source of stress for students. Similarly, Indian Bachelor of Physiotherapy students [21] reported that unpredictability of examinations and fear of failure were sources of stress, which align with responses generated from this study.

Placement related stressors have been frequently identified across timepoints 1 and 3. Student responses were commonly related to professionalism, with stress arising when not given immediate feedback from clinical educators and having to complete self-directed learning and professional development tasks. Additionally, financial pressures arose as a theme throughout this study, and was amplified during the clinical placement block. Students often had to travel, therefore increasing accommodation costs, as well as finding difficulty to attain or maintain part time positions. Hamshire et al. [47] found that negative placement experiences have a significant impact with the potential for becoming the ‘tipping point’ for students. This study by Hamshire et al. [47] proposed a variety of suggestions for avoiding attrition, which include flexible start and finish times, maximum travelling distances to placement, central feedback database, and student financial support funds. Similarly, a Dutch study [5] on six undergraduate students conducted across three universities found placement stressors had an impact on the student’s well-being. Some stressors included demanding supervisors, worrying about not being good enough, and worrying about meeting expectations. Potentially, students are being taught the appropriate clinical and academic skills but are not being taught appropriate professional skills. Students may benefit from skills in taking on feedback and understanding the importance of reflective practice. If expectations of clinical placements are clearly defined prior, students may have reduced stress or better coping mechanisms.

The second research question focused on the influence of sex in relation to stress and burnout throughout the first year of the program. Whilst the current study did not identify sex to influence stress or burnout, there were unique differences across all three timepoints in the first year, and female students self-reported experiencing more stress. The sex difference has previously been reported, with females experiencing more stress than males [21] but not in relation to burnout in physiotherapy students [24, 35] or qualified physiotherapists [30, 32, 48]. A cross-sectional study completed on 231 undergraduate students in Pakistan reported that not only did women differ in their perception of stressors, but their reactions also differed, where they were more overtly reactive [21]. It is explained this could be due to the gender role socialization of emotions, or that females are possibly more emotionally responsive. This cross-sectional study may have differing experiences of stress for females due to a variety of factors including the length of the study, the age of the participants, or the outcome measure utilised (Student Life Stress Inventory) [21].

The third research question concentrated on coping mechanisms utilized by DPHTY students during the first year of their program. Qualitative themes from open-ended questions revealed a mix of maladaptive and positive coping strategies. Previously, a cross-sectional study reported [35] that three general strategies exist in an attempt to cope with stressful situations: problem-focused coping, emotion-focused coping, and avoidance-focused coping. The questionnaire did not require the DPHTY students to identify whether they were receiving any external mentoring or support for any psychological illness. Thus, it is not possible to rule out whether any of the participants at each timepoint were diagnosed with any psychological illness. Within our study, students reported that they wish they had been made aware of and equipped with effective coping strategies to overcome such stressors related to the demands of the program. The organizing theme, course survival tips, illustrated that students felt unprepared for the course demands, and desired more self-management strategies when stressful periods arise.

The most common problem-focused coping strategy reported in the current study was exercise, with reports of it being a beneficial stress reliever across all three time-points. Whilst the use of exercise as a coping strategy is potentially not surprising for a physiotherapy cohort, such strategies should be promoted due to its proven stress-relieving benefits [12, 49]. For university students, opportunities to engage in sporting and social clubs often incur no additional costs, potentially promoting accessibility. This may be of benefit for HEIs; not only encouraging health and well-being, but also allowing for social interaction outside of class time. Students reported that maintaining social relationships within and outside of the program have been an effective emotion-focused coping strategy in managing stress. These findings concur with previous studies [4, 11] where maintaining relationships are important for coping. Being able to debrief and have open conversations appears to be imperative to the well-being of students.

Although the majority of students reportedly utilize positive coping strategies, there were also accounts of maladaptive coping strategies. The avoidance-focused coping strategies that have been identified as maladaptive include alcohol consumption, excessive eating, and gaming. Maladaptive coping strategies found by a cross-sectional American University study [10] included alcohol abuse, drug abuse, and smoking. They further recommended that universities provide ongoing research outlining stress, how to detect when an individual may be experiencing high levels of stress, and positive strategies to deal with such stressors. Perhaps in future, HEIs could increase wellness education and services such as advisement and counselling. Students may benefit from group discussion around health and wellbeing, and the challenging aspects around maladaptive coping strategies. This environment may elicit more students to speak when feeling stressed or burnt out.

The findings from our study offer unique insights around the pressures and expectations of pre-registration physiotherapy students. Responses provide information to aid in the maintenance of student well-being, whilst ensuring production of quality health professionals, that may be of benefit to HEIs. It is important for physiotherapy programs to know the causes of stress and burnout in their students, and associated coping mechanisms utilised. Findings from previous studies [47, 50] in which students also report burden with travel for placement, financial costs, inflexible timetabling, and clinical placement stressors, demonstrate commonality with this research. It would be valuable to determine the ‘tipping point’ [47] for students undertaking pre-registration physiotherapy programs and trial targeted intervention strategies to mitigate potential stress and burnout.

### Strengths and limitations

To our knowledge, this is the first study to explore both perceived stress and burnout in postgraduate DPHTY students. This study aims to serve as a foundation for future guidance and to inform HEIs program structure to optimize student welfare, academic ability, and support. This paper also allows for increased understanding of how an intensive course can impact the welfare of students. The use of previously validated and reliable tools coupled with open-ended questions allowed for a broad understanding of the complexity of being a student in an intensive pre-registration, postgraduate DPHTY program. Quantitative and qualitative data gathered has allowed for the research questions to be answered with a humanistic view. The use of two reliable and valid questionnaires (CSE and MBI-GS (S)), further enhances the strength of this study and allows for confidence in results generated.

The authors acknowledge methodological limitations identified as the students’ lack of understanding of the true definition of burnout. This was recognised as students reported feeling burnt out in the open-ended questions however did not meet the criteria according to the Maslach Burnout Inventory [41, 43]. This was discovered as a potential threat after the implementation of the survey. It is recommended that future studies provide key definitions within surveys to avoid misinterpretation of stress and or burnout by participants. Another methodological limitation included the length of time conducted, as well as a relatively small sample size. Whilst this study focussed on three key transitional timepoints in the first year of the DPHTY program, it is not possible to transfer these findings across the entire 2-year program.

Research has shown that culture can influence burnout [51]. As such, different cultural backgrounds among the students may present as different attitudes towards stress, burnout and study requirements and serve to confound these results. Future studies should explore whether cultural backgrounds and values have any impact on stress, burnout and coping mechanisms used by pre-registration physiotherapy students.

## Conclusion

In summary, this study has presented a unique insight into experiences, perceptions, and behaviours that DPHTY physiotherapy students possess in response to perceived stress, burnout, and coping strategies. This study has demonstrated that the DPTHY students reportedly experience varying levels of stress and burnout throughout their first-year program. Students consistently exemplified high levels of stress within the context of coursework and academic load. Although not significant, female students, generally, tended to report higher levels of perceived stress across all three timepoints. There were no differences between the sexes in regard to self-reported burnout. Whilst the relationship between stress and burnout was explored, no strong correlations were found. This study has also identified that physiotherapy students were implementing coping strategies, both positive and maladaptive and highlights the importance of continued research within postgraduate, pre-registration physiotherapy programs to identify why students may be experiencing stress and burnout and to guide positive rather than maladaptive coping strategies. With further research, HEIs may implement frameworks to identify student stressors and burnout, adapt their program structure, and implement management strategies for students. Further exploration of optimal coping strategies to mitigate and manage perceived academic stress and burnout within the first year of an intensive DPHTY Physiotherapy program is warranted. Findings of the current study provide valuable insights to inform curriculum design and optimize the transition to intensive academic study and pre-placement support for DPHTY physiotherapy students.

## Data Availability

The datasets used and/or analysed during the current study are available from the corresponding author on reasonable request. Permission was obtained from Mind Garden, Inc. on 26 June 2019 to reproduce copies of the Maslach Burnout Inventory™ Instruments and Scoring Keys for the purpose of this study. MBI - General Survey for Students - MBI-GS (S): Copyright©1996, 2016 Wilmar B. Schaufeli, Michael P. Leiter, Christina Maslach & Susan E. Jackson. All rights reserved in all media. Published by Mind Garden, Inc., www.mindgarden.com

## Abbreviations

AVG: Average
CSE: Coping self efficacy
CYN: Cynicism
DPHTY: Doctor of Physiotherapy
EXH: Exhaustion
HEI: Higher educational institutions
MBI-GS(S): Maslach Burnout Inventory- General Survey for Students
PE: Professional efficacy
SAF: Social and Academic Functioning
T1: Timepoint one
T2: Timepoint two
T3: Timepoint three
